# Immune Responses against Disseminated Tumor Cells

**DOI:** 10.3390/cancers13112515

**Published:** 2021-05-21

**Authors:** Ling Peng, Yongchang Zhang, Zibing Wang

**Affiliations:** 1 Department of Pulmonary and Critical Care Medicine, Zhejiang Provincial People’s Hospital, Hangzhou 310014, China; 2 Lung Cancer and Gastrointestinal Unit, Department of Medical Oncology, Hunan Cancer Hospital, The Affiliated Cancer Hospital of Xiangya School of Medicine, Changsha 410013, China; zhangyongchang@csu.edu.cn; 3 Department of Immunotherapy, Affiliated Cancer Hospital of Zhengzhou University & Henan Cancer Hospital, Zhengzhou 450008, China

**Keywords:** immune response, metastases, immune surveillance, immunotherapy, disseminated cancer cells

## Abstract

**Simple Summary:**

Metastasis in general represents the progression phenotype whereby cancer cells break from a malignant primary location and travel to and invade other distant organs. Theoretically, tumor cells that exit the primary tumor might be eliminated by immune cells. The immune system has the ability to recognize and eliminate malignant tumor cells; however, failed immune surveillance contributes to cancer development. Disseminated tumor cells persist and reemerge as a clinically symptomatic disease. How do disseminated tumor cells evade immune surveillance? In this context, tumor/immune system interactions play a key role and are the subject of intense scrutiny. After colonization, the immunosuppressive tumor microenvironments of metastatic lesions promote tumor growth and worsen prognosis. Here, we discuss scientific advances relating to the interaction between disseminated tumor cells and the immune cells in tissue-specific tumor microenvironments.

**Abstract:**

Most cancer-related deaths are a consequence of metastases, a series of linear events, notably the invasion–metastasis cascade. The current understanding of cancer immune surveillance derives from studies in primary tumors, but disseminated cancer cells acquire mutations and, in some cases, appear to progress independently after spreading from primary sites. An early step in this process is micrometastatic dissemination. As such, the equilibrium between the immune system and disseminated cancer cells controls the fate of the cancer. Immune checkpoint inhibitors (ICIs) exhibit significant clinical activity in patients, but the efficacy of ICIs depends on both the tumor and its microenvironment. Data often suggest that disseminated cancer cells are not adequately targeted by the immune system. In this review, we summarize the main basic findings of immune responses against disseminated tumor cells and their organ-specific characteristics. Such studies may provide new directions for cancer immune therapy.

## 1. Introduction

Metastasis is often a sluggish process, with fewer than 0.01% of cells typically surviving to reach another organ [1]. Cancer cells that metastasize to a distant organ are expected by some to be therapy-resistant populations. The inability of current anticancer therapies to target dormant disseminated tumor cells (DTCs) yields a specific clinical challenge, simply highlighted by the worse survival rate of all disseminated solid malignant tumors. Various immune cells work together to affect metastatic outgrowth. Multiple immune cell populations promote metastasis by establishing an immunosuppressive microenvironment or by conditioning the premetastatic niche. Cancer cells, however, undergo immune surveillance by both the innate immune system and adaptive immune system, and each participates in affecting cancer cell fate [2,3].

Primary tumors reduce the exposure and access between cancer cells and antitumor immune systems. The tumor microenvironment (TME) typically has an immunosuppressive status that inhibits the induction of effective antitumor immunity. Given these challenges, cancer immunotherapy has not achieved its full potential yet for treating large primary tumors and poorly immunogenic tumors. The immunotherapeutic effect of immune checkpoint inhibitors (ICIs) depends on the TME, which is constituted by different cell types [4]. Growing evidence indicates that the TME of the primary tumor facilitates disseminated tumors to show off dormant or proliferative states in metastatic organs. Thus, understanding the organ-specific response of the tumor–immune system interaction is central to future directions in this regard.

## 2. Immune-Mediated Metastatic Growth on Metastatic Dormancy

**Figure 1:**
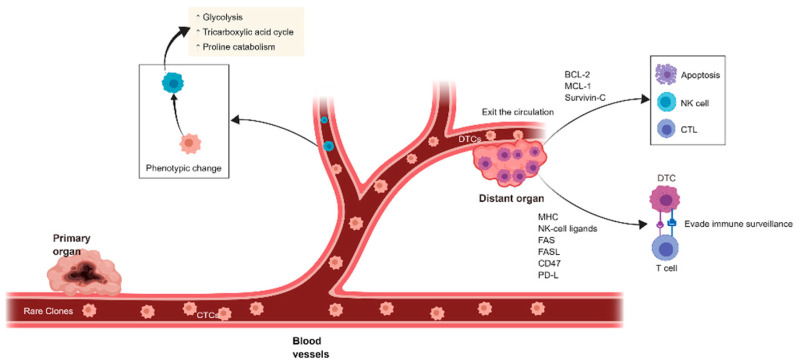
**Cancer metastatic cascade involving CTCs and DTCs**. Tumor cell dissemination facilitates the cancer cell to spread from its site of origin. Key events of the cascade are outlined. Changes in cellular properties are necessary to allow the development of an invasive phenotype, including increased glycolysis, tricarboxylic acid cycle, and proline catabolism. DTCs obtain the ability to resist NK cell or CTL-mediated killing by upregulating antiapoptosis molecules, including BCL-2, MCL-1, and survivin-C. DTCs dysregulate the expression of MHC molecules and immune checkpoint molecules (CD47, CD155 and PD-L1) to evade immune surveillance.

Cancer cell dormancy includes quiescent and slowly dividing cancer cells [5]. Circulating tumor cells (CTCs) can be monitored in the peripheral circulation in the early stages of malignant disease [6]. After CTCs implant on target organs, they are named disseminated tumor cells (DTCs) [7]. Therefore, dormant cancer cells can be found as CTCs in the peripheral blood or as DTCs within tissues, and typically fail to respond to conventional anticancer therapy [8]. The biologic characteristics and the interaction between CTCs and other cells in these elusive and multistep processes are regulated by many cancer-promoting molecules (Figure 1). For example, during the process of dissemination, pancreatic and bile duct cancer CTCs form immune-evasive multi-ingredient cell clusters in the peripheral circulation, indicating a continuous immune evasion status that promotes CTC proliferation [9].

Tumor cells may disseminate throughout the body even before the primary tumor can be detected. Tumor cells obtain the ability to escape immune system attacks, then survive and outgrow in distant organs. Single DTCs differ from micrometastasis in that the latter are comprised of >20-cell clusters [3]. As micrometastatic dissemination is an early event, the destiny of DTCs is unclear. DTCs can shirk the microenvironment or therapy-induced stresses, ultimately becoming detectable metastatic lesions [10]. In some cases, inflammatory responses and immune cells support spreading and metastasis [11]. Whether immune microenvironments can detect dormant DTCs remains an unanswered question. Mouse experiments indicate that tumor cells can be continuously controlled by CD8^+^ T cells in the bone marrow [12], which can also supply immune surveillance [13]. This immune equilibrium is also maintained by CD4^+^ T cells and cytokines, including interferon-γ and interleukin-12.

Supportive metastatic niches provide a microenvironment enabling disseminated cancer cells to successfully metastasize [14]. Therefore, targeting the metastatic niche is a promising method to eradicate dormant cells [15]. Dormant cancer cells can, however, reawaken in response to signals, resulting in recurrence and metastasis. In the process of metastasis, DTCs obtain the ability to be resistant to natural killer cells or cytotoxic T cell-mediated killing by upregulating antiapoptosis molecules, including BCL-2, MCL-1, and survivin-C [16]. DTCs may evade immune surveillance through dysregulation of the expression of MHC I and MHC II molecules, NK cell ligands, and immune-checkpoint molecules (CD47, CD155 and PD-L1, etc.); thus, DTCs can persist for an extended period [17].

Metastatic progression often proceeds from rare clones in primary tumors [18]. Cancer cell plasticity makes these cells less immunogenic. The immune system eliminates more immunogenic cancer cells through cancer immune editing. Tumor heterogeneity is in turn related to immune infiltration in cancer [19]. In microenvironments, programs of dormancy in DTCs are activated in a complex manner [20]. In an established congenic tumor cell clone library derived from primary mouse pancreatic adenocarcinoma, transcriptomic and epigenetic analyses indicated that each tumor elicits unique immune infiltrations correlating with therapeutic responses [21]. Single-cell sequencing results have verified that tumors are constituted by a diverse array of heterogeneous tumor cells, fibroblasts, and immune components [22]. In the tumor microenvironment, the distribution of immune cell subsets is spatially specific in different locations. Spatial and functional heterogeneity of immune cells in the tumor immune microenvironment, together with other factors, drive immunological heterogeneity.

Signaling networks and gene expression patterns are dysregulated when the metabolism pattern is changed [23]. Metabolic plasticity significantly affects the survival of cancer cells in the metastatic process. Once CTCs emerge in the circulation before locating the metastatic site, they must alter their metabolism to adapt to the new environment of the target organs. Differences in CTC preferences for specific organs can be partially attributed to their metabolic properties [24]. Depending on the metastatic site, tumor cells increase their energy requirement via amplifying the signal activity of critical ATP-producing pathways, including glycolysis and reactive oxygen metabolism, and unusual metabolism pathways, such as proline catabolism, also participate in this process [25]. This is one example of many we could provide.

## 3. Immune Cell Population Composition in Immune Response

**Figure 2:**
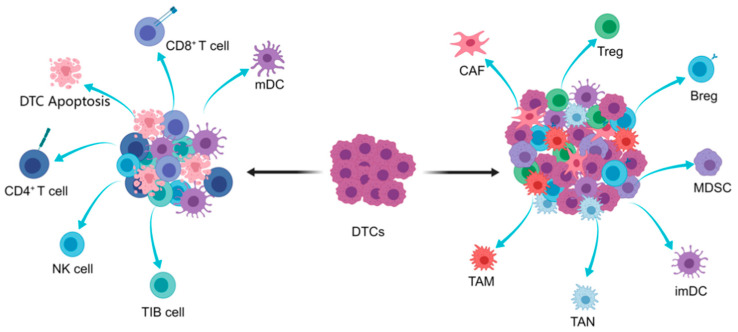
**The interaction of immune cells and DTCs**. As disseminated tumor cells extravasate, they encounter a foreign microenvironment with obstacles to survival. During this process, cellular components play key roles in the destiny of DTCs. Numerous interactions between cell types are involved throughout tumor progression and metastasis. If the microenvironment is filled with cytotoxic CD8^+^ T cells, mature DCs, and NK cells, DTCs will thus go into apoptosis (**left**). DTCs survive and proliferate within immunosuppressive microenvironments containing CAFs, TAMs, TANs, MDSCs, etc. (**right**).

Research on immune evasion provides detailed insights into molecular mechanisms of tumor development and metastasis [26]. Crosstalk between cancer cells and auxiliary cell energy sources promote tumor progression. Immune cells include myeloid-derived inhibitory cells participating in the regulation of innate immunity, and lymphoid cells that participate in the regulation of adaptive immunity. The other immunological cells are involved in pathogen defense. Immune cells are phenotypically plastic and thus can be “re-programmed” or “educated” to provide protumor immune effects. Tumor cells have obtained multiple molecular mechanisms to avoid immune cells’ attacks in peripheral circulation. Tumor fate is determined by the balance between tolerogenic and effector immune response. The role of immune cells in DTC dormancy has been confirmed in immune-deficient nude mice models with spontaneous lung metastasis [27,28]. Most reports focus on the metastasis-promoting role of immune cells—for example, tumor-associated macrophages (TAMs) and metastasis-associated macrophages (MAMs)—through influencing the multiple steps of cancer metastasis [3]. An effective immune response must turn over the functions of TAMs plasmacytoid dendritic cells, neutrophils, Tregs, and Bregs to inhibit cytotoxic tumor lymphocyte (CTL) and/or NK cell infiltration, proliferation, and immune surveillance functions (Figure 2).

### 3.1. Innate Immune Response

- Dendritic cells (DCs)

Dendritic cells (DCs) function as essential antigen-presenting cells (APCs) involved in multiple biological processes [29]. Based on different biological functions, DCs are classified into plasmacytoid DCs (pDCs) and “classical” or “myeloid” DCs. DCs are a class of vital immune cells, representing a critical bond between adaptive and innate immunity [30]. Thus, the function of DC exhaustion plays a vital role in antigen-specific immune evasion. Immature DCs (imDCs) take up and process antigens and present antigenic peptides on MHC molecules [31]. The accumulation of imDCs leads to decreased immune surveillance [32]. When trended by chemokine and infiltrating into tumors or migrating to lymphatic organs, mature DCs (mDCs) promote antigen presentation and activate cytotoxic T lymphocytes (CTLs). Tumor-infiltrating DCs are present in many cancer types [33]. The infiltration of mDCs in tumors is associated with the increased recruitment of immune effector cells [34]. Cancer-derived granulocyte colony-stimulating factor (G-CSF) leads to a systemic inhibition in DCs [35]. Tumor cell-derived IL-6 and vascular endothelial growth factor (VEGF) also affect the differentiation and maturation of DCs [36]. Melanoma cells secrete CCL4, which attracts conventional DC type 1 (cDC1) and can be blocked by β-catenin signaling, indicating that the inhibition of tumor DC recruitment may be a dominant mechanism in tumor intrinsic β-catenin activation [37].

- Natural killer (NK) cells

As innate immune cells, natural killer (NK) cells participate in eliminating infected or transformed cells. NK cells recognize and eliminate tumor cells either directly or depend on engaging NK cell CD16 receptor molecules by antibody-bound tumor cells [38]. DTCs evade the NK cells’ attack in primary tumors, circulation, and metastatic sites by upregulating the expression of inhibitory ligands. Malignant cells are exempt from NK cell recognition and elimination via complicated mechanisms [39]. NK cells exert an antimetastatic response; therefore, whether metastatic cancer cells can avoid NK cell recognition is crucial to dissemination. The receptor repertoires of NK cells can be differentially regulated by cancer cells [40]. Experimental studies reveal that DTCs are exempt from immune elimination of NK cells by the downregulation of multiple activation receptors on the NK cell surface [41]. These results highlight the critical function of NK cells in inhibiting tumor progression. NK cells are promising candidates for immunotherapy given that decreased MHC-I molecule expression is one of the important mechanisms of immune evasion [42]. Monoclonal antibodies blocking NK cell inhibitory receptors—for example, NKG2A and KIR—can enhance NK cell-mediated cytotoxicity [43,44].

### 3.2. Adaptive Immune Response

- T cells

T cells encompass complicated subsets that take part in lymphomagenesis, including memory T cells, Tregs, and naive T cells [45]. Tumor-infiltrating lymphocytes (TILs) participate in antitumor immunity in the lung tumor niche, and increased infiltration of TILs correlates with a better prognosis in several solid cancers [46]. Activated CD8^+^ T cells, γδ-T cells, and CD4^+^ Th1 cells are important in regulating type I immune responses, while Th2, Tregs, and Th17 are frequently associated with cancer progression and a worse prognosis [47]. DCs effectively acquire, process, and present tumor-associated antigens (TAAs) participating in activating the antitumor function of CD8^+^ T cells. Immunologically ‘hot’ tumors, such as melanoma and non-small-cell lung cancer (NSCLC), demonstrate an abundance of T cell infiltration. The antitumor immunity of CD8^+^ T cells may effectively eliminate DTCs [28]. By rejuvenating the endogenous antitumor function of T cells during tumor progression, ICIs exert therapeutic activity against a variety of cancer types. The T cell-induced gene expression profiles of ‘hot’ tumors are associated with a higher response rate to ICI immunotherapies [48]. However, immune-tolerant T cells assist tumor cells to adapt to the tumor microenvironment and facilitate cancer inflammation. Along with T cell dysfunction, the apoptosis of T cells has been confirmed as a potential mechanism of tumor resistance to immunotherapy [49]. Tumor rejection mediated by tissue-resident memory CD8^+^ T (Trm) cells sparks the cascade delivery of CTL responses via dermal DCs [50]. A protective subgroup of the CD8^+^ T cell population recently identified with CD39^+^PD-1^+^CD8^+^ T cells correlates with prolonged disease-free survival after resection [51]. CD4^+^CD25^+^ regulatory T cells decrease the antitumor immune response and inhibit the curative effect of cancer immunotherapies. Tregs are widely considered to be protumorigenic, as they express inhibitory cytokines and immune checkpoint molecules which inhibit CD4^+^ and CD8^+^ T cell function [52]. Tregs are selectively trended into tumor tissues by tumor cells in a CCL22- and CCL28-dependent manner; subsequent Treg-induced secretion of VEGF-A by cancer cells promotes endothelial cell proliferation [53]. Local immunomodulation of Treg cell depletion can eradicate tumor cells at distant sites [54].

- B cells

B cells constitute a critical portion of TILs in several cancers. They modulate immune responses by secreting antibodies, delivering antigens, and interplaying with other cells in TME. Previous studies reported that B cells play a critical role in regulating the function of T cells, including T cell activation, proliferation, and the formation of memory T cells [55]. Tumor-infiltrating B lymphocytes (TIBs) exist in all stages of human lung cancer during the disease progression [56]. TIBs are mainly positioned at the lymphoid aggregates in lung tumors and named as tertiary lymphoid structures (TLS) [57]. TIBs preserve the function and structure of the TLS in the lung TME by secreting cytokines and chemokines. Activated B cells can also directly attack tumor cells via releasing the granzyme B and the cytokine TRAIL [58].

Regulatory B cells (Bregs) are a subgroup of B cells with the function of promoting tumor progression. Memory CD27^+^ and transitional CD38^+^ B cells are canonical characteristics of Bregs [59]. Bregs execute their immunoregulatory functions by releasing inhibitory cytokine and intercellular communication. Bregs have a distinct function in attenuating antitumor response, namely, releasing anti-inflammatory mediators, such as IL-10 and TGF-β, which induce T cell differentiation to Tregs [60]. The effects of Bregs include modulating antitumor response through directly inhibiting the function of effector T cells [61]. Bregs inhibit immune responses via regulating intercellular immune checkpoint molecular interaction: for example, CD40/CD40L, Fas/FasL, and CTLA-4/CD86 [62,63]. Human CD19^+^CD25^hi^ Bregs can also strengthen the biological function of Tregs [64].

### 3.3. Immunosuppressive Cell Subsets

- Tumor-associated macrophages (TAMs)

Based on the biological function, activated macrophages were classified as proinflammatory (M1 type) or anti-inflammatory (M2 type or TAMs). High-grade tumor-associated macrophages (TAMs) are correlated with a worse prognosis and decreased overall survival in xenograft and cancer patients [65]. TAMs can secrete IL-1β expression via the WNT pathway, promoting IL-17 secretion by activated γδ T cells. This increases systemic G-CSF, which in turn facilitates DTC proliferation and pulmonary metastases [26]. TAMs can also promote tumor progression by fostering angiogenesis, stimulating cancer cell proliferation, remodeling the extracellular matrix, accelerating metastasis, and expediting the functional exhaustion of antitumor effector immune cells. Even before dissemination, TAMs induce protein expression, which promotes a pro-37 dissemination (Mena^INV^) and pro-dormancy (NR2F1) phenotype in the tumors [66]. Cancer cells and stroma can also secret chemokine ligand 2 (CCL-2) to recruit C-C chemokine receptor type 2 (CCR2^+^) monocytes in metastatic lesion, facilitating tumor seeding [67]. Furthermore, TAMs can foster a fibrotic microenvironment with increased endothelial permeability, thus nurturing colony development of disseminated cancer cells [68]. In a genetically engineered breast cancer mouse model, Prune-1 expression augmented the M2 polarization of TAMs through TGF-β enhancement and IL-17F secretion, thus facilitating lung metastasis [69].

- Tumor-associated neutrophils (TANs)

Neutrophils are one of the most common myeloid cell subtypes and the earliest immune cells to be recruited to injury tissue. Neutrophils may be the initial cells in the lung premetastatic niche [70]. As the innate immune system to eliminate pathogens, neutrophils are quickly activated in response to intrusive tumor cells. Based on their functional heterogeneity, the two polarization states of TANs, “N1” and “N2”, have been described, although their distinction remains disputed [71]. N1-neutrophils express antitumor cytokines (TNF-α, IL-12, etc.), while N2-neutrophils express proangiogenic and immunosuppressive cytokines (VEGF, TGF-β1, etc.) [72]. Tumor-associated neutrophils (TANs) are associated with a worse prognosis in several malignancies. TAN recruitment to the TME is mainly induced by CXCR2 ligands and TGF-β [73]. Furthermore, neutrophils promote breast cancer cell metastasis and colonization in the lung by inhibiting CD8^+^ T effector cells [74]. TANs induce T cell exhaustion by facilitating tumor-derived granulocyte macrophage colony-stimulating factor-mediated PD-L1 upregulation [75]. TANs can initiate cancer metastasis through MAC-1/ICAM-1 axis-mediated cell-to-cell communication between TANs and cancer cells [76]. However, results regarding the role of TANs are conflicting, and the dual biological function of TANs in promoting and suppressing the cancer of tumor cells remains controversial [77]. In human colorectal cancer, increased CD66b^+^ TANs in tumors enhance the tumoricidal capacity of CD8^+^ T cells and are associated with better prognosis. Neutrophils inhibit intraluminal NK-mediated cancer cell elimination and enhance the extravasation of metastatic malignant cells [78]. Breast cancer cells can accelerate neutrophils to comprise metastasis-supporting neutrophil extracellular traps (NETs) [79]. Premetastatic lung cancer shows a high infiltration of cytotoxic neutrophils that prevent tumor cell seeding in a niche with low TGFβ activity [80].

- Myeloid-derived suppressor cells (MDSC)

Myeloid-derived suppressor cells (MDSC) are a class of a heterogeneous subtype of immature myeloid cells that accumulate in tumor-bearing hosts. By promoting TIL exhaustion, MDSC demonstrate significant features for inhibiting the immune response [81]. One of the main mediators, ARG1, is a pivotal enzyme for the urea cycle and maintains an immunosuppressive microenvironment by the depletion of L-arginine, subsequently blockading T cell infiltration [82]. MDSC also expresses inducible nitric oxide synthase (iNOS), which can catabolize L-arginine to induce T cell anergy [83]. MDSC characteristic molecular markers include CD11b and Gr-1, CD11b^+^Ly6C^low^Ly6G^+^ cells ranging as a granulocytic subset, while CD11b^+^Ly6C^high^Ly6G^−^ classified as a monocytic subset [84]. The immunologic landscape and TME vary among organs and this discretely shapes MDSC repertoires [85]. In liver and lung metastasis, pSTAT3 and pSTAT5 signaling, respectively, exert dominant effects on MDSC programming, indicating that MDSC programming as a response to malignant tumors is highly dependent upon organ-specific conditions and is adaptable. Macrophage depletion effectively reduces the CSC fraction, sensitizing them to chemotherapy in vivo [86].

- Cancer-associated fibroblasts (CAFs)

Fibroblasts, normally found in the connective tissue, regulate tissue remodeling during wound healing and development. They are also a major cell type in the tumor stroma. Disseminated breast cancer cells arouse adaptive characteristic changes in lung fibroblasts by secreting interleukin-1α (IL-1α) and IL-1β, forming a supportive metastatic niche [87]. Moreover, immune cell-derived IL-1β accelerates nuclear factor-κB (NF-κB) activation in fibroblasts [88]. CAFs are highly heterogeneous in tumor tissues. Among the different CAF populations, the immunosuppressive role of FAP+ CAFs has been explored [89]. As a heterogenous group of mesenchymal cells, CAFs grafted with breast carcinoma cells enhance tumor formation in mice [90]. CAF-directed cancer invasion is seen in a zebrafish xenograft, while prostate and colorectal cancer-derived fibroblasts facilitate metastasis during the early stage of these malignant diseases [91]. CAFs persistently receive and/or respond to stimuli, affecting other immune cells in the TME [92]. CAFs regulate the outgrowth of dormant metastatic CSCs by modulating their metabolism [93].

## 4. Metastatic Organ Differences in Immune Characteristics

**Figure 3:**
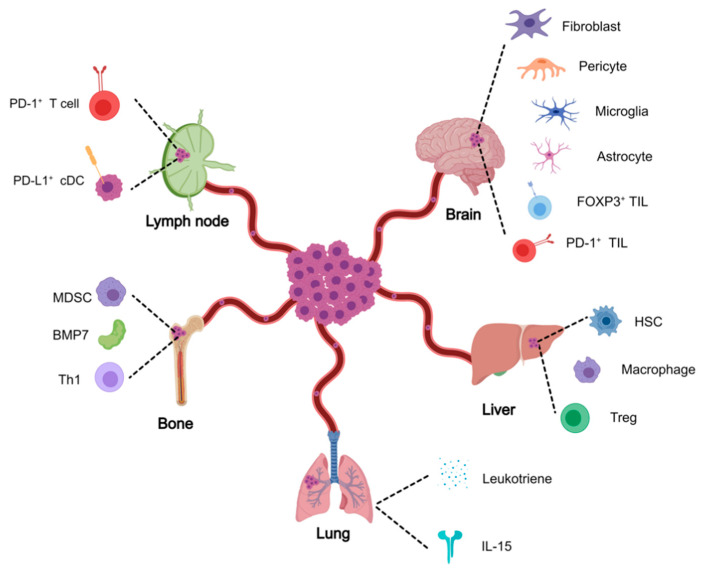
**Colonization of DTCs in different organs**. Disseminating cells selectively colonize in different tissues and commence the process of further dissemination. The expansion to a metastatic colony relies on the ability to initiate organ-specific colonization programs that allow the tumor cells to survive in a new microenvironment. Colonization is dependent upon a combination of tumor cell and tissue-specific factors. Description of organ-specific metastatic programs is described in the text.

Whether a primary cancer cell forms a colonized lesion depends on the ability to survive within the circulation system and specific organs. Organ-specific growth of malignant tumors is an adaptation of the selection and growth process (Figure 3). Host organs are not passive receivers of CTCs; instead, they are actively and selectively modified by the primary tumor before metastatic spread. Understanding organ-specific mechanisms which enable metastatic growth is of great importance. Some cancers primarily spread to one specific organ or show sequential organ-specific colonization. Different tumor types exhibit significant variability in their metastatic route (by the varied length of latency periods), the organs affected, and the type of metastasis. The immune responses of the primary tumor versus the metastatic sites vary depending on the organs involved. Experimental animal models have revealed tumor extrinsic and intrinsic mechanisms that dictate organ-specific metastasis against massive attrition of DTCs [94]. The quest to find supportive niches is key for the survival of DTCs [14]. Cancer stem cells preferentially upregulate PD-L1 expression, and thus, ICIs can potentially eradicate disseminated stem cells [95].

The pattern of metastatic organs varies significantly depending on tumor types [96]. Variation in pretreatment infiltration of immune cells may lead to differential activity of nivolumab depending on the metastatic organ. In addition, the anatomy of vessels and organ-specific circulation patterns influence metastatic spread [97]. Krausgruber et al. showed that organ-specific and cell-type-specific differences in immune gene activity are reflected in the patterns of chromatin regulation [98]. Tumor-immune microenvironments of different organs and gene expression of various tumors may influence immune responses to checkpoint inhibitors [99].

### 4.1. Organ-Specific Responses

The dissemination process depends on extrinsic factors, such as vascular wall accessibility and circulation patterns, as well as on the innate capacities of the metastatic tumor cells [100]. As types of foreign invaders may vary by anatomic location, the immune system has evolved to customize host defenses accordingly. Published research provides a greater focus on how the fate of DTC is influenced by specific organs, with less emphasis on the mechanisms of primary tumor dormancy [101].The tissue-specific biology in the TMEs contributes to different therapeutic responses. Furthermore, distinct TIMEs (tumor immune microenvironments) can coexist within an individual patient [99]. Tumor cells require similar molecular profiles to escape immune surveillance and grow in a secondary niche, regardless of their origin. The anatomy of vessels and organ-specific circulation patterns influence metastatic spread [97]. Different organ sites have distinctive immune microenvironments typified by the presence of tissue-resident innate immune cells [102]. Multiomic profiling and integrative bioinformatic analysis of structural cells, including fibroblasts, endothelium, and epithelium are important contributors to our understanding of immune responses [98].

Multiple checkpoint pathways regulate T cell activation at different stages in tumor immunology. Central to this process are programmed cell death 1 (PD-1) and cytotoxic T-lymphocyte-associated antigen 4 (CLA-4) immune checkpoint pathways. PD-1 and CTLA-4 pathways operate at different stages of an immune response [103]. Studies investigating whether responses of different metastasis sites vary suggest that site-specific metastasis exhibits differential responsiveness to ICI therapy [104]. Meta-analysis based on clinical trial data also indicates that different metastatic sites have varied responses to ICIs [105]. Based on clinical observations, “organ-specific response criteria” adapted from RECIST 1.1 and irRECIST (immune-related RECIST) are used to evaluate ICI response [106].

### 4.2. Lymph Nodes

An immune response induced by ICI treatment is mostly observed in tumor-draining lymph nodes (TDLNs) [107]. A study that retrospectively analyzed CT scans of patients with metastatic NSCLC receiving nivolumab found that treatment was more significant in the lymph nodes compared to other organs such as the bone, adrenals, and liver [108]. Mouse tumor models show that TDLNs are abundant with tumor-specific PD-1^+^ T cells that strongly associate with PD-L1^+^ cDCs [109]. However, removing TDLNs concurrently with primary tumors does not affect the response to ICIs on secondary tumors due to the immunotolerance in TDLNs [110]. PD-L1 expression of lymph node metastases specimens is low and thus inadequate to guide ICI treatment in clinical practice [111]. PD-1 expression on CD3^+^ T cells is significantly increased in the metastatic lymph nodes of NSCLC [112]. On the other hand, studies investigating benign regional lymph nodes have found morphological differences with unique immune cell populations observed across responders and non-responders to immunotherapy [113].

### 4.3. The Brain

Historically, the brain was considered an immune suppressive microenvironment [114]. The PD-L1 expression of brain metastases is relatively low, which may be related to the immune sanctuary features of the brain [111]. The TME of the brain consists of multiple types of cells, including microglia, pericytes, fibroblasts, astrocytes, and a variety of suppressive or stimulatory immune cells [115]. It is unclear whether adaptive immune cells exert an antimetastatic response within the brain parenchyma or in perivascular spaces. Preclinical findings suggest that T cell priming in the extracranial compartment is essential for an effective immune response in the CNS [116]. That immune surveillance in brain metastasis shares similarities with that in extracranial tumors argues for research to investigate the role of ICIs for the treatment of solid tumor brain metastasis. The efficacy of ICIs for brain metastases are similar in ICI monotherapy and combination regimens, indicating that PD-(L)1 inhibition has similar activity inside and outside the brain. Genome-wide sequencing analyses of metastatic variants have identified MYC as a crucial regulator for the adaptation of disseminated tumor cells to the activated brain microenvironment [117]. Infiltration of FOXP3^+^ regulatory TILs and exhausted PD-1^+^ TILs have been observed in brain metastasis specimens [118]. Epigenetic modeling also contributes to the immune-suppressive tumor and the profile of the brain metastasis TME [119]. Retrospective studies and prospective trials with immune ICIs demonstrate that the brain can harbor an “active” immune microenvironment for immunotherapy [120].

### 4.4. The Liver

The liver is a distinct organ with an immunosuppressive environment. Circulating metastatic tumor cells that reach the liver are faced with unique cellular populations. Liver parenchyma is abundant with cells of the innate immune system that can be obstacles to cancer cells, including Kupffer, stellate, the sinusoidal endothelium, and inflammatory cells that are mediated through cell–extracellular matrix adhesion [121]. Hepatic stellate cells (HSCs) can release chemokines and cytokines to recruit immune cells, thus shaping the immune microenvironment [122]. HSCs orchestrate a prometastatic niche following tumor extravasation. Furthermore, macrophages, hepatocytes, and liver sinusoidal endothelial cells contribute to this process by releasing TNFα and TGFβ [123]. Metastasis to the liver remains a therapeutic challenge for ICIs, as confirmed in a mouse model where liver metastasis triggered apoptosis of tumor-specific T cells [124]. Liver metastases limit the efficacy of immunotherapy via T cell elimination mediated by macrophages. Using a mouse model of dual tumor immunocompetency, it is found that immune response in the presence of tumor antigen within the liver leads to immune suppression. The immune suppression is associated with the Tregs activation and modulation of intratumoral CD11b^+^ monocytes [125]. ICIs are less effective in cancer patients with liver metastasis [126]. However, due to the limited number of studies and patients, it is still controversial whether patients with liver metastases would benefit less from ICIs compared with other metastatic sites. A survival benefit was not observed with ICIs simply with chemotherapy, unless in combination with anti-VEGF therapy, indicating the role of angiogenesis blockade with ICIs [105].

### 4.5. The Lung

The lungs are the most common metastatic site for a variety of cancers. CTCs shed from primary tumors follow a systemic path and reach the lungs, where blood is oxygenated. Lungs thus appear as the first host for DTCs, and the lungs are the mostly seeded organ during metastasis. Cells that do not metastasize to the lungs are then recirculated to remote organs via the arterial system, leading to a wider distribution of metastasis [127]. Lung capillaries are equipped with a basement membrane and the mediators of tumor extravasation in the lung have been identified (e.g., COX2, MMP2 and SPARC) [100,128]. Cytokines are essential in sculpting the tumor microenvironment, and various studies investigated the role of cytokines in promoting lung metastasis. In an animal tumor model, neutrophils propagate metastasis-initiating cancer cells via the secretion of leukotrienes, thus facilitating lung metastasis [70]. Using heterotopic and intravenous injection models of lung metastasis of mice, interleukin-5 (IL-5) was found to be a pivotal factor in metastatic colonization in lungs through the regulation of immune cells in the microenvironment of the distal lung [129]. Based on different immune statuses, metastatic samples can be organized into three immune clusters [128]. Compared with other metastatic sites (brain, liver, or bone), metastasis to the lung presents with a higher immunogenic score. In hepatocellular carcinoma, lung metastases respond most favorably to ICIs in terms of objective response rate [106].

### 4.6. Bones

Bone metastasis is an adverse predictor the for efficacy of ICIs in clinical scenarios [129,130]. Specific cellular and molecular niches in the bone microenvironment may impact tumor-to-bone metastasis, including multiple immune cell types [131]. Bone tissues have less effective cytotoxic cells and a large amount of suppressor immune cells [132]. Therefore, PD-L1 expression in bone metastases is less important to the process of immune escape. In bone marrow, immature myeloid cells differentiating into MDSCs may acquire immunosuppressive activity [133]. Other factors, such as bone morphogenetic protein 7 (BMP7), can induce dormancy of prostate cancer cells [134]. An animal model of bone metastasis of breast cancer indicates that tumor cells become integrated into the bone matrix shortly after reaching the bone, but that only a minority reach the bone marrow [135]. During metastasis to bone, cancer cells acquire a mesenchymal phenotype to facilitate immune escape and dissemination [136]. Analysis of bone marrow samples of prostate cancer patients reveals a lack of Th1 cells in the tumor; high levels of TGF-β in prostate cancer bone metastases constrain the Th1 lineage, which confers resistance to ICI therapy [137]. These data emphasize the significance of the organ-specific niche in dictating differences in T cell lineages.

## 5. Conclusions

This review identifies the abundance of work still to be done and the many challenges that remain for clinical practice. Immune responses toward disseminated tumor cells during cancer metastasis are complex and dynamic processes. The distinct TME at metastatic organs interacts with the adaptive immune system to determine responses to immunotherapy, which is partly mediated by the resident innate immune cells. Interpatient differences in the TME and organ-specific responses influence the duration and type of responses to ICIs. More effort toward understanding immune surveillance mechanisms in different metastasis sites, including the brain, bone, and liver, is needed.

## Data Availability

Data sharing not applicable.
